# Associations of childhood hearing loss and adverse childhood experiences in deaf adults

**DOI:** 10.1371/journal.pone.0287024

**Published:** 2023-06-21

**Authors:** Wyatte C. Hall, Timothy D. V. Dye, Shazia Siddiqi

**Affiliations:** 1 Public Health Sciences, University of Rochester Medical Center, Rochester, New York, United States of America; 2 Pediatrics, University of Rochester Medical Center, Rochester, New York, United States of America; 3 Obstetrics & Gynecology, University of Rochester Medical Center, Rochester, New York, United States of America; 4 Neurology, University of Rochester Medical Center, Rochester, New York, United States of America; 5 Center for Community Health and Prevention, University of Rochester Medical Center, Rochester, New York, United States of America; Örebro University Faculty of Medicine and Health: Orebro universitet Fakulteten for medicin och halsa, SWEDEN

## Abstract

Childhood trauma and adverse childhood experiences have a strong relationship with health disparities across the lifespan. Despite experiencing approximately doubled rates of trauma, Adverse Childhood Experiences (ACEs) are poorly characterized in deaf populations. We sought to characterize deaf-specific demographic factors and their association with multiple experiences of ACEs before the age of 18 years old. An analytical cross-sectional approach was used to ascertain associations of deaf-specific demographic factors and experiences with ACEs. The complete dataset included 520 participants for a total response rate of 56%. After adjusting for confounding effects, less severe hearing loss of 16–55 dB (2+ OR: 5.2, 4+ OR: 4.7), having a cochlear implant (2+ OR: 2.1, 4+ OR: 2.6), and not attending at least one school with signing access (2+ OR: 2.4, 4+ OR: 3.7) were significantly and independently associated with reported experiences of multiple ACEs. We conclude that factors associated with childhood hearing loss and language experiences increase risk of experiencing ACEs. Given the strong relationship between ACEs and poor social outcomes, early intervention clinical practice and health policies should consider interventions to support healthy home environments for deaf children.

## Introduction

Over the past decades, childhood maltreatment and trauma have been linked to increased risk of adverse adult health outcomes as signified by the 1998 CDC-Kaiser Adverse Childhood Experiences (ACEs) study [1]. Generally, higher incidence of ACEs is associated with increased risk in poor health behaviors, and worse mental and physical health in adulthood [2]. ACEs also have a dose effect where multiple experiences lead to worse outcomes [3]. ACE-associated consequences are experienced as early as adolescence [4] and, among others, includes increased risk of premature death [5] and allostatic changes [6], and impacts the healthy development of future generations [7]. Most recently, ACEs are described as a “major public health problem in the United States” worsening during the COVID-19 pandemic [8]. One population especially vulnerable to childhood trauma, and therefore ACEs, are children with pre-lingual hearing loss.

Rates of various trauma experiences are nearly doubled in the deaf population (see review in Johnson et al. [9]). A study of childhood abuse and neglect found 76% of deaf college students reported such experiences, and were significantly more likely than hearing college students to experience emotional and physical neglect and abuse, including sexual abuse [10]. Those who experience childhood sexual trauma are highly likely to be re-victimized in adulthood [11]. Additionally, there are unique deaf traumas such as poor communication with hearing and non-signing parents [12, 13], and “information deprivation trauma” where already-traumatic life events are intensified due to limited information access (such as natural disasters and family deaths) [14]. Unsurprisingly, the deaf population also experiences significant health disparities. Apart from well-documented language, cognitive, and education disparities, the deaf population experiences less access to language-concordant preventive services, decreased health literacy, increased use of the emergency room, and increased obesity, suicide, and interpersonal violence, among others [15–18].

The elevated prevalence of trauma and disparities occurs in a context of the majority (90–95%) of deaf children being born into hearing families that have previously never met a deaf person, and do not know a natural sign language such as American Sign Language (ASL) [19]. The mismatch between the typical spoken language of the home and the child’s inability to effortlessly access spoken language can create a neurodevelopmental emergency, given language acquisition’s foundational role in healthy brain and overall human development [20–22].

The population of children with hearing loss can be quite heterogeneous in terms of the type and severity of hearing loss, language exposure, and assistive technology use. For those with severe enough hearing loss, language development is immediately at risk if they do not receive effortless access and exposure to language in their daily environment, creating cognitive, socioemotional, and physical developmental consequences that are not fully ameliorated by hearing loss technology (such as cochlear implants) [23]. For some, this phenomenon is increasingly described as “language deprivation” [20, 24] in deaf communities, and can become severe enough to be its own mental health syndrome in adulthood [25]. Everyday communication barriers, social isolation in the home, and risk of delayed development make deaf children susceptible to both typical and unique forms of abuse and neglect. In fact, preliminary theorizing of explanatory factors underlying the general population’s adult health consequences of ACEs included language acquisition disruptions, and highlighted deaf children as a salient example where such a relationship may exist [26].

Only two known studies have explored ACEs in deaf communities. A study of 376 deaf Norwegians found an association between three ACEs–physical abuse by parent (36%), peer bullying (23%), and serious sexual abuse (30%)–and adult mental health problems (30%) [27]. Another study proposed “Adverse Childhood Communication Experiences” (ACCEs) which is poor direct and indirect childhood communication with parents and other family members. ACCEs were associated with increased risk of diabetes, hypertension, heart disease, lung disease, depression, and anxiety in a sample of 1,524 adults who reported becoming deaf before 13 years old [28].

To date, ACEs are an understudied topic in the deaf population (for instance, the largest prevalence estimate of ACEs to date used a telephone survey which excluded this population [3]). Since deaf children can experience entirely unique and complex childhood contexts (including hearing loss severity, sign language use, the influence of cochlear implants, and various educational environments, among others) relative to the general population, we sought to characterize what deaf-specific demographic factors, if any, may elucidate increased risk for experiencing ACEs before the age of 18 years old.

## Methods

### Study design

An analytical cross-sectional approach combining two similar studies (due to deaf communities generally being difficult to recruit large samples from) was used to ascertain associations of Adverse Childhood Experiences (ACEs) in deaf populations. The study design included a comprehensive list of standard and deaf-specific demographic factors alongside 10 ACE questions over two phases of data collection.

#### Study 1 (“Adverse childhood experiences pilot project”)

The University of Rochester Research Subjects Review Board (UR RSRB) determined this study (UR RSRB STUDY00003417) met federal and university criteria for exemption with the need for written consent waived. Inclusion criteria included self-reported childhood deafness, age 18 years or older, and being able and willing to consent to participate in the study. Exclusion criteria included no hearing loss, under 18 years of age, and those who were not able to consent and participate in the study. Data collection began May 2019 and concluded January 2020. In total, 148 people identifying as d/Deaf and two as Deafblind participated.

#### Study 2 (“Developing a measure of deaf childhood experiences”)

The UR RSRB determined this study (UR RSRB STUDY00003417) met federal and university criteria for exemption with the need for written consent waived. Inclusion criteria included self-reported childhood deafness, born and/or raised in the USA (before age five), age 18 years or older, and being able and willing to consent to participate in the study. Exclusion criteria included no hearing loss, raised in the USA after five years old, under 18 years of age, and those who were not able to consent to participate in the study. Data collection began April 2020 and concluded July 2020. In total, 355 people identifying as d/Deaf, and 115 as Deafblind participated.

### Recruitment

Community recruitment for both studies occurred on social media platforms commonly used by deaf people (Facebook and Twitter), person-to-person networking, and through community organizations. Two of the authors (WCH and SS) are deaf and have community networks that were used for recruitment as well. Participants were routed to online REDCap surveys for participation.

### Study variables

General and deaf-specific demographic factors, and self-reported ACEs (recategorized as having experience 0 or 1, 2+, and 4+) were the study variables. General demographics included age, race and ethnicity, household income, education attainment, employment status, sexual orientation, and gender. Deaf-specific demographics included self-reported hearing loss status (slight, mild, moderate, moderately-severe, severe, profound), hearing loss onset (birth, before 3, 3–5, 5+), parent hearing status (at least one deaf parent, hearing), education setting (at least one oral-only setting, at least one setting with signing access), cochlear implant status (yes, no), cochlear implant surgery age (0-11m, 1-1y11m, 2-2y11m, 3-3y11m, 4-4y11m, 5+ years), current use of the cochlear implant (never, sometimes, most of the time, all the time), and signing exposure before five years old (yes, no). Survey questions sought to capture granular complexity of certain demographic factors for other project needs. For the purposes of these analyses, however, demographic variables were collapsed into simplified logical constructs where possible and appropriate. The study language was written English and both studies ascertained variables in the same manner. IRB approval was obtained to combine datasets and remove duplicates (i.e., participants who participated in both studies).

#### Data cleaning and quality

Data quality was addressed through implementing required responses, skip patterns, and range checking in implementation of the REDCap surveys. As a result, question-specific missing data rates were minimal (generally <1%) and out-of-range values were prevented. Several demographic variables (sexual orientation and race, for example) were collected as open-ended responses to prioritize respondent self-classification and those responses were subsequently recoded into discrete categories. Cronbach’s alpha for the ACEs scale used in this study was 0.734, which met or exceeded Cronbach’s alpha levels for other ACEs studies [29].

### Analysis

The main study outcome was self-reports of experiencing two or more (2+) ACEs [27], and four or more (4+) ACEs [2]. Hosmer and Lameshow’s approach to logistic regression [30] was used by crosstabulating demographic variables with the main outcomes to generate bivariate associations. Any variables demonstrating at least marginal statistical association with the main outcome (p<0.10) were included in the subsequent multivariate model. All marginally-significant demographic variables were subsequently entered into a forward-stepwise logistic multivariate regression, with any significant variables (p<0.05) remaining in the final model after controlling for other variables. The Goodness-of-Fit statistic evaluated how well the data was fit. SPSS v28 (IBM Corporation) was used for all analyses.

## Results

A total of 926 respondents across both studies initiated the survey on REDCap. 520 participants formed the complete dataset after filtering incomplete responses (190), duplicates (29, via email address), and those who did not self-identify as deaf, Deaf, or Deafblind (187) for a total response rate of 56%. In terms of standard demographics, the participant sample was primarily aged 25–34 years (58%), white (77.8%), straight (76.1%), female (53.2%), and earning $35,000 to $74,999 (combined 59.1%) with a college degree/some graduate school (33.1%) while being employed full-time (60.3%). For deaf-specific demographics, the participant sample reported hearing loss onset mainly at birth (34.3%), identifying as Deaf (62.2%), having hearing parent(s)/guardian(s) (71.5%), experiencing at least one oral-only setting (54.5%), one education placement (61.7%), being exposed to some type of signing before five years old (59.5%) and having a cochlear implant (50.9%). Of those who identified having a cochlear implant, most had the surgery at five years or older (43%), and currently use the implant either sometimes or most of the time (combined 82%).

Bivariate associations of demographic variables with the presence of 2+ and 4+ ACEs are presented in Table 1. For general demographic factors, only age under 35 years was significantly associated with both 2+ and 4+ ACEs, and race other than white was marginally-significantly associated. No other general demographic variables were significantly associated. In contrast, multiple deaf-related demographic factors were significantly associated with both 2+ and 4+ ACEs including: (1) slight-to-moderate hearing loss, (2) not attending at least one school with signing access, and (3) having a cochlear implant.

**Table 1:** Bivariate associations of general and deaf-related variables with presence of 2+ and 4+ adverse childhood experiences.

Variable	ACEs		Odds Ratio (95% Confidence Interval)	p-value	ACEs		Odds Ratio (95% Confidence Interval)	p-value
	Two or more present % (n)	Zero or one present % (n)			Four or more present % (n)	Zero to three present % (n)		
**Demographic Variables**								
**Age**								
<35 years	80.2 (324)	19.8 (80)	3.2 (2.1, 5.0)	<0.001	66.1 (267)	33.9 (137)	5.5 (3.5, 8.8)	<0.001
35 or more years	55.7 (64)	44.3 (51)	Referent		26.1 (30)	73.9 (85)	Referent	
**Race**								
Other than white	81.7 (94)	18.3 (21)	1.7 (1.0, 2.8)	0.051	65.2 (75)	34.8 (40)	1.5 (1.0, 2.4)	0.050
White	72.8 (294)	27.2 (110)	Referent		55.0 (222)	45.0 (182)	Referent	
**Income**								
<$50,000	77.2 (217)	22.8(64)	1.3 (0.9, 2.0)	0.160	57.7 (162)	42.3 (119)	1.0 (0.7, 1.5)	0.831
$50,000 or more	71.8 (171)	28.2 (67)	Referent		56.7 (135)	43.3 (103)	Referent	
**Education**								
High school or less	81.1 (60)	18.9 (14)	1.5 (0.8, 2.8)	0.212	52.7 (39)	47.3 (35)	0.8 (0.5, 1.3)	0.320
College or higher	74.3 (318)	25.7 (110)	Referent		58.9 (252)	41.1 (176)	Referent	
**Employment**								
Full-Time	75.7 (237)	24.3 (76)	1.1 (0.8, 1.7)	0.535	59.1 (185)	40.9 (128)	1.2 (0.9, 1.7)	0.286
Other than Full-Time	73.3 (151)	26.7 (55)	Referent		54.4 (112)	45.6 (94)	Referent	
**Gender**								
Other than male/female	90.0 (9)	10.0 (1)	-	0.512	60.0 (6)	40.0 (4)	-	0.257
Male	75.0 (174)	25.0 (58)			54.0 (149)	46.0 (127)		
Female	73.9 (204)	26.1 (72)			61.2 (142)	38.8 (90)		
**Sexual Orientation**								
Other than heterosexual	80.6 (100)	19.4 (24)	1.5 (0.9, 2.5)	0.084	62.1 (77)	37.9 (47)	1.3 (0.9, 2.0)	0.209
Heterosexual	72.9 (288)	27.1 (107)			55.7 (220)	44.3 (175)	Referent	38.8 (90)
**Deaf-related Variables**								
**Deaf Identity**								
DeafBlind	87.5 (84)	12.5 (12)	2.7 (1.4, 5.2)	0.001	65.6 (63)	34.4 (33)	1.5 (1.0, 2.4)	0.065
Deaf/deaf	71.9 (304)	28.1 (119)	Referent		55.3 (234)	44.7 (189)	Referent	
**Hearing Loss Status**								
Slight (16–25 dB) to moderate loss (41–55 dB)	94.8 (73)	5.2 (4)	5.8 (2.1, 16.2)	<0.001	88.3 (68)	11.7 (9)	5.5 (2.7, 11.3)	<0.001
Moderately severe (56–70 dB) to profound (91+dB)	76.0 (291)	24.0 (92)	Referent		58.0 (222)	42.0 (161)	Referent	
**Age of Hearing Loss Onset**								
Age five or older	88.9 (72)	11.1 (9)	2.4 (1.5, 5.0)	0.017	79.0 (64)	21.0 (17)	2.5 (1.4, 4.5)	0.001
Under five	77.0 (292)	23.0 (87)	Referent		59.6 (226)	40.4 (153)	Referent	
**Parents’ Hearing Status**								
Both parents are hearing	76.5 (284)	23.5 (87)	1.4 (0.9, 2.1)	0.137	59.6 (221)	40.5 (150)	1.4 (1.0, 2.0)	0.088
One or both parents are deaf	70.3 (104)	29.7 (44)	Referent		51.4 (76)	48.6 (72)	Referent	
**Attended at Least One Oral-only School**								
Yes, attended oral-only school	82.2 (88)	17.8 (19)	1.7 (1.0, 3.0)	0.045	59.8 (64)	40.2 (43)	1.1 (0.7, 1.8)	0.544
No, did not attend oral-only school	72.8 (300)	27.2 (112)	Referent		56.6 (233)	43.4 (179)	Referent	
**Attended at Least One School with Signing Access**								
No, did not attend at least one school with signing access	80.2 (329)	19.8 (81)	3.4 (2.2, 5.4)	<0.001	65.1 (267)	34.9 (143)	4.9 (3.1, 7.8)	<0.001
Yes, attended at least one school with signing access	54.1 (59)	45.9 (50)	Referent		27.5 (30)	72.5 (79(	Referent	
**Received a Cochlear Implant**								
Yes, received a cochlear implant	86.0 (227)	14.0 (37)	3.6 (2.3, 5.5)	<0.001	73.5 (194)	26.5 (70)	4.1 (2.8, 5.9)	<0.001
No, did not receive a cochlear implant	63.1 (161)	36.9 (94)			40.4 (103)	59.6 (152)	Referent	
**Hearing Aid Use**								
Yes, ever	74.5 (313)	25.5 (107)	0.9 (0.6, 1.6)	0.800	57.4 (241)	42.6 (179)	1.0 (0.7, 1.6)	0.883
No, never	75.8 (75)	24.2 (24)	Referent		56.6 (56)	43.4 (43)	Referent	
**Signed Before Age Five**								
No, did not sign before age five	75.7 (159)	24.3 (51)	1.1 (0.7,l 1.6)	0.680	55.2 (116)	44.8 (94)	0.9 (0.6, 1.2)	0.451
Yes, signed before age five	74.1 (229)	25.9 (80)	Referent		58.6 (181)	41.4 (128)	Referent	

Table 2 presents the results of the multivariate model. Participants with slight-to-moderate hearing loss, who did not attend at least one school with signing access, and who had received a cochlear implant were all significantly more likely to report 2+ and 4+ ACEs after adjusting for confounding effects. Participants who self-identified as Deafblind were more likely to report 2+ ACEs while those with hearing loss onset after the age of five years old were more likely to report 4+ ACEs. The Pearson Goodness-of-Fit statistic was not significant (not shown), indicating the data fit the model well.

**Table 2:** Multivariate forward stepwise logistic regression model of 2+ and 4+ adverse childhood experiences.

Variable in model	Odds Ratio, 2+ ACEs (95% Confidence Interval)	p-value	Odds Ratio, 4+ ACEs (95% Confidence Interval)	p-value
Slight (16–25 dB) to moderate loss (41–55 dB)	5.2 (1.8, 15.0)	0.002	4.7 (2.1, 10.3)	< .001
Did not attend at least one school with signing access	2.4 (1.4, 4.1)	0.002	3.7 (2.1, 6.4)	< .001
Received a cochlear implant	2.1 (1.2, 3.5)	0.005	2.6 (1.7, 4.1)	< .001
Age of hearing loss onset after five years old	(not in model)		2.1 (1.1., 3.9)	0.02
Identify as DeafBlind	2.1 (1.0, 4.1)	0.040	(not in model)	
Sexual orientation other than heterosexual	2.2 (1.2, 4.2)	0.019	2.2 (1.3, 4.0)	< .001
Age less than 35 years	1.9 (1.1, 3.4)	0.030	3.3 (1.9, 5.9)	< .001

Additional variables entered into model that did not remain significant: Attended oral-only school, race other than white

## Discussion

This study is the first-ever characterization of deaf-specific demographic factors that may increase risk of reporting Adverse Childhood Experiences (ACEs). Previous work has indicated that experiencing two or more (2+) ACEs was significantly associated with reported mental health issues for deaf individuals [27], and four or more (4+) ACEs was considered high risk for toxic stress physiology in the general population [2]. Several factors remained significant predictors of reporting both 2+ and 4+ ACEs in our multivariate models, even after controlling for the confounding effects of other variables. These factors were (1) less severe hearing loss (16–55 dB), (2) having a cochlear implant, and (3) not having attended at least one school with signing access.

Detecting less severe hearing loss as a significant factor in our multivariate models was unexpected, as much of the literature for deaf trauma is centered around profoundly deaf individuals and signing communities without considering the circumstances around hearing ability. It is unclear what this association may mean for deaf vulnerability to trauma (other than the already-acknowleged heightened vulnerability that deaf children experience). For example, a study asking mental health providers who work with deaf communities about protective factors against trauma highlights access to information, language, and communication, but does not consider hearing loss severity and the use of technology [9]. The same is true for other highly-cited studies that have discussed trauma in deaf communities [10, 11, 14].

Concurrently, the literature on cochlear implants and subsequent socioemotional development generally focuses on studies where adults report about a deaf child (e.g., parents, teachers, and other individuals) rather than asking the deaf child directly [31–34]. While understanding adult perspectives about deaf children with whom they interact is important–when primary reports do not derive from the deaf child personally, negative experiences could be underreported or overlooked. Additionally, 43% of cochlear implant recipients in our sample had the implant inserted after five years old–relatively late for neurolinguistic development. Now, children more commonly experience cochlear implant surgery as early as one year of age (or earlier). It is unclear if implantation age may have an impact on the relationship with ACEs (for instance if earlier implantation led to cochlear implant outcomes that better aligned with family expectations) and deserves further investigation. Determining whether or not cochlear implants are protective in deaf childhood trauma is unclear; our study suggests this is an important area to investigate further.

In contrast, the controversy of signed language use in early child development and education settings for deaf children is strongly documented and a long-running thread in deaf communities’ discourse and advocacy stretching back at least several centuries [21, 35]. This discouragement of signed language use by medical and education systems has led to a landscape where many deaf children are placed in public school settings (and specialized schools) that do not include signed languages and visual communication strategies. Our study reflects this experience since a majority of our participants have experienced an oral-only school environment. Importantly, “signing access” was defined very broadly within the analyses as long as some signing was present in their school environment. This included any kind of access such as signing communication systems (such as Signed Exact English), and a full range of settings from having just an interpreter in a public school setting to attending a fully immersive residential school using American Sign Language. That not attending a school with some sort of signing access was a risk factor suggests that visual languages and communication may have a protective role for reducing risk of ACEs.

Taken as a whole, these predictive factors related to hearing status and school settings may indicate pressure to “pass for hearing” [36–38] in deaf children who–either individually or through some combination–have some usable residual hearing, who acquired some usable hearing through the cochlear implant in the hearing family home, and/or who were not placed in a signing educational environment. The majority of medical and educational interventions for deaf children are typically centered around restoring as much hearing as possible, discouraging sign language use, and ensuring as much English fluency and speech clarity as possible [39]. Such scenarios can also reflect a complex picture that may include parent decision-making values, their attitudes about hearing loss and disability, the types of early intervention services families receive and types of local educational settings that are available, socioeconomic status, and much more. What this overall picture may mean exactly in terms of heightened vulnerability to ACEs requires and deserves more investigation in future work.

Because hearing loss can only ever be remediated to the functional limits of hearing loss technology (hearing aids and cochlear implants) [24], deaf and hard of hearing children still experience the impact of hearing loss and many may fail to “pass for hearing.” Indeed, in our sample, even with 50% having cochlear implants and 54.5% experiencing a school setting without sign language access, a majority (62.2%) still identified with a culturally Deaf label that signifies membership and participation in signing deaf communities. Over time, continual failure to “pass for hearing” as deaf children continue to develop throughout their childhood may lead to accumulating and ongoing strains in family and social dynamics.

There is also the well-known context of the extra family stress that can occur when a child has a disability, and specifically hearing loss [40]. Generally, parents with children who have cochlear implants experience more stress than those with non-disabled children [41]. The experience of a dual diagnosis for Deafblind children can create additional pressure for families beyond typical stress of a disability diagnosis where parents especially need support for their mental health, advocacy skills, and learning how to parent a Deafblind child [42]. Overall, individual and societial expectations from and for both the hearing family and the deaf child to be “normal” may lead to a home environment that is more conducive to stress and negative experiences, contributing to trauma experiences of deaf people.

Our study has limitations, and caution should be taken in interpretation and generalizability of these results. The online survey was in written English, a second language for many members of the deaf population; additionally, language fluency in ASL and English was not directly assessed. The retrospective nature of asking adults about ACEs may introduce recall bias. Online surveys carry risk of false responses, multiple responses by the same person, and skewed data based on recruitment channels. The sample was predominantly white, preventing analyses exploring additional racial and ethnic intersectionalities. Furthermore, those with unilateral hearing loss is a population of interest that should be included in future work. Our sample demographics are heavily educated, possibly a function of the research team being situated in Rochester, NY where there is a large, highly-educated culturally Deaf population.

More attention should be given toward deaf children with multiple disabilities and hard-of-hearing children who are frequently “stuck in the middle” as they have usable (albeit limited) hearing, often appearing to be “hearing” with their listening and speaking abilities. Likewise, those with other marginalized identities (such as sexual orientation) need support as well. For families that have a child experience hearing loss several years after birth who are already developing spoken language, there should be sufficient support and counseling to address what may be a sudden and drastic developmental change with their child. Additionally, more consideration is needed as to what role the cochlear implant plays in the family home and how it may alter family dynamics, expectations, and interactions with the deaf child. Overall, it is important that future investigations explore the intersectionalities of deaf individuals with multiple marginalized identities and how this may influence the risk for ACEs.

### Public health implications

Overall, there is emerging attention on the relationship between childhood experiences and adult health outcomes in the deaf population. This interest includes increasing community and academic dialogue about language deprivation and its downstream effects across the lifespan [13, 20, 25], which this study appears to indicate also includes a heightened risk of ACEs. Such heightened risk aligns with calls for bimodal bilingual education as a public health measure [43] to improve deaf population health. Given the strong relationship between adverse childhood experiences and poor health and social outcomes across the lifespan, changes in clinical practice and health policies in early intervention, and education systems, and the medical home should be considered that better supports healthy and safe home environments for deaf children.
